# Optical coherence tomography angiography findings in patients with ocular and non-ocular Behcet disease

**DOI:** 10.5935/0004-2749.20210031

**Published:** 2021

**Authors:** Pinar Topcu Yilmaz, E.Yildiz Ozdemir, Mehmet Numan Alp

**Affiliations:** 1 Health Sciences University, Ankara Numune Education and Research Hospital, Ankara, Turkey

**Keywords:** Angiography, Behcet syndrome, Fovea centralis/blood supply, Tomography, optical coherence, Uveitis, Angiography, Síndrome de Behçet, Fóvea central/ irrigação sanguínea, Tomografia de coerência óptica, Uveites

## Abstract

**Purposes:**

To evaluate the optical coherence tomography angiography findings in patients with Behçet disease with and without ocular involvement.

**Methods:**

A total of 40 patients with Behçet disease and 30 healthy controls were enrolled in the study. Retinal vessel density in the superficial capillary plexus and deep capillary plexus, foveal avascular zone area and perimeter, acirculatory index, foveal density, and nonflow area in the superficial retina were automatically measured using the optical coherence tomography angiography software AngioVue and compared between the groups.

**Results:**

The mean parafoveal and perifoveal vessel densities in the superficial capillary plexus and deep capillary plexus and foveal density were significantly lower in the eyes with Behçet uveitis compared to the eyes without Behçet uveitis and eyes of the healthy controls. In the eyes with Behçet uveitis, logMAR visual acuity showed a moderate correlation with parafoveal and perifoveal vessel densities and foveal density (r=-0.43, p=0.006; r=-0.62, p<0.001; r=-0.42, p=0.008; respectively).

**Conclusion:**

Behçet disease with posterior uveitis was associated with significant perifoveal and parafoveal vascular decrements in the superficial and deep retina.

## Abstract (pt)

**Objetivo:**

Avaliar achados de angiografia por tomografia de coerência óptica em pacientes com doença de Behçet com e sem acometimento ocular.

**Métodos:**

Foram incluídos 40 pacientes com doença de Behçet e 30 controles saudáveis. A densidade vascular retiniana nos plexos capilares superficial e profundo, a zona avascular foveal, o índice de circularidade, a densidade foveal e a área sem fluxo da retina superficial foram medidos automaticamente, através do software *AngioVue* para angiografia por tomografia de coerência óptica, e comparados entre os grupos.

**Resultados:**

A densidade vascular parafoveal e perifoveal média nos plexos capilares superficial e profundo, bem como a densidade foveal, foram significativamente menores nos olhos com uveíte de Behçet em comparação com os olhos sem uveíte de Behçet e os olhos dos controles saudáveis. Nos olhos com uveíte de Behçet, a acuidade visual logMAR mostrou correlação moderada com a densidade vascular parafoveal e perifoveal e com a densidade foveal (respectivamente, r=-0,43, p=0,006; r=-0,62, p<0,001; e r=-0,42, p = 0,008).

**Conclusão:**

A doença de Behçet com uveíte posterior foi associada a decréscimos significativos da vascularização perifoveal e parafoveal na retina superficial e profunda.

## INTRODUCTION

Behçet disease is a chronic, multisystemic, autoinflammatory disease that is highly prevalent in countries along the ancient Silk Road. It is characterized by immune-mediated vasculitis that can affect blood vessels of any size in all organ systems. Ocular inflammation is most commonly in the form of relapsing, remitting uveitis and develops in >70% of patients^(1)^.

The current gold standard for documentation and monitoring of posterior segment involvement in Behçet disease is fluorescein angiography as it can easily detect retinal vascular leakage, retinal ischemia, and macular edema^(2)^. However, it is an invasive procedure, requiring injection of an exogenous dye, and limited in its depth resolution^(2,3)^.

Optical coherence tomography angiography (OCT-A) has recently become an excellent tool to obtain high resolution en face images of the retinal and choroidal microvasculature and analyze changes in the superficial capillary plexus (SCP), deep capillary plexus (DCP), and choriocapillaris separately^(4)^. Both qualitative and quantitative changes within the retinal microvasculature can be assessed with OCT-A. Although its role in the evaluation of retinal vascular diseases, such as diabetic retinopathy, retinal venous occlusions, retinal arterial occlusions, and age-related macular degeneration, has been extensively examined in the recent literature, few studies have investigated the use of OCT-A in Behçet uveitis^(5-12)^.

The present study aimed to evaluate the quantitative OCT-A findings in patients with Behçet disease with and without uveitis and compare these findings with those in healthy controls.

## METHODS

This cross-sectional, observational study was conducted at Ankara Numune Training and Research Hospital, Turkey. The study protocol was approved by the Institutional Review Board and adhered to the tenets of the Declaration of Helsinki. Informed consent was obtained from all subjects before participation in the study.

Patients who fulfilled the diagnostic criteria of the International Study Group for Behçet’s Disease were included in the study between February 2018 and December 2018. Patients with other retinal and/or optic nerve diseases (diabetic retinopathy, hypertensive retinopathy, central serous chorioretinopathy, macular degeneration, glaucoma, optic neuropathy, etc.), cystoid macular edema (CME), refractive errors > ±3.0 diopters of spherical equivalence, history of ocular trauma or surgery within the previous 3 months, and systemic comorbidity that could affect the retinal microvasculature, such as diabetes mellitus and hypertension, were excluded. The patient group was divided into two subgroups. Patients without any evidence of active or inactive anterior or posterior uveitis were included in the non-ocular Behçet group and patients with a history of posterior uveitis or active posterior uveitis related to Behçet disease were included in the ocular Behçet group. The control group consisted of healthy subjects who visited our outpatient clinic for refractive error <3.0 D of spherical equivalence.

All participants underwent a detailed ophthalmological examination including measurement of best corrected visual acuity (BCVA), slit-lamp biomicroscopy, tonometry, and a detailed fundus examination. Fundus photography and fluorescein angiography were used to evaluate posterior uveitis.

OCT-A images were obtained with RTVue-XR Avanti (AngioVue, Optovue, Inc., Fremont, CA, USA), which operates via the split spectrum amplitude decorrelation angiography algorithm and motion correlation technology built into the AngioVue system. A 6 × 6-mm frame centered on the fovea was scanned. After image acquisition, en face OCT angiograms were segmented to define the superficial and deep layers using the built-in automatic segmentation algorithm of the device^(13)^. Eyes with a central macular thickness ≥300 µm, eyes with poor quality scans with a signal strength index of <40, and eyes with unreliable measurements due to extensive disruption of the microvascular structure were excluded from the analysis.

The newly developed AngioAnalytics software (version 2017.1.0.151) was used to obtain measurements of vessel density (VD) in the SCP and DCP, foveal avascular zone (FAZ) area, FAZ perimeter, acirculatory index (AI), foveal VD 300 µm around the FAZ (FD-300) in the total retina, and nonflow area in the superficial retina and compare the measurements with those of the healthy controls^(14)^. If both eyes were eligible for the study, the data collected from the eye with better image quality were used in the analysis.

The statistical analyses were performed using SPSS version 17.0 (Chicago, IL, USA). The normality of the data was determined by Shapiro-Wilk test. The Kruskal-Wallis test was used to compare the OCT-A parameters among patients with Behçet disease with ocular involvement, patients with Behçet disease without ocular involvement, and control subjects. The categorical variables were analyzed using Pearson’s X^2^ test, and Pearson correlation analysis was used for correlative analyses. Quantitative variables were presented as mean ± standard deviation. A p-value <0.05 was considered statistically significant, except in subgroup analyses requiring Bonferroni correction, in which appropriate adjustments were made.

## RESULTS

A total of 20 patients with Behçet uveitis, 20 patients with Behçet disease without ocular involvement, and 30 healthy controls were included in this study. The groups were similar with respect to age and sex. The clinical characteristics of the study groups are summarized in table 1.

**Table 1:** Clinical characteristics in patients with Behçet disease and healthy controls

	BD with posterior uveitis	BD without ocular involvement	Control	p-value
Age (years)	36 ± 12.2	40.1 ± 10.7	39.6 ± 9.6	0.1^*^
Male/female	9/11	4/16	13/17	0.2^*^
BCVA (logMAR)	0.2 ± 0.3	0.01 ± 0.05	0 ± 0	<0.001^*^
Central macular thickness (µm)	240.2 ± 46	245.9 ± 29.2	248.8 ± 20.7	0.9^*^
Disease duration (years)	10.9 ± 7	9.7 ± 7.3	NA	0.7^**^

* = p-value for Kruskal-Wallis test;

** = p-value for Mann-Whitney U test.

BD= Behçet disease; BCVA= best corrected visual acuity; NA= not applicable.

The foveal VDs in both the SCP and DCP were similar among the groups (p=0.9, p=0.6; respectively). The parafoveal and perifoveal VDs in the SCP and DCP were significantly reduced in patients with Behçet uveitis. FD-300 was also significantly reduced in the Behçet uveitis group. There was no significant difference in FAZ area, FAZ perimeter, AI, or nonflow area measurements among the three cohorts (Table 2).

**Table 2:** Comparison of OCTA measurements in eyes with and without Behcet uveitis and healthy controls

	Patients with Behcet disease with posterior uveitis (n=20)	Patients with Behcet disease without ocular involvement (n=20)	Control	p-value ^*^	p-value^1^	p-value^2^
Superficial foveal VD (%)	20.1 ± 7.3	18.9 ± 9.9	19.5 ± 9.4	0.82	NA	NA
Superficial parafoveal VD (%)	41.7 ± 6.9	47.3 ± 4.4	47.9 ± 7.2	0.002	0.005	0.02
Superficial perifoveal VD (%)	42.5 ± 5.5	46.8 ± 3.0	48.1 ± 3.6	0.001	<0.001	0.005
Deep foveal VD (%)	32.8 ± 8.9	34.5 ± 10.0	35.5 ± 8.5	0.36	NA	NA
Deep parafoveal VD (%)	47.2 ± 6.3	52.7 ± 3.7	52.9 ± 4.2	0.001	<0.001	0.002
Deep perifoveal VD (%)	41.4 ± 5.1	46.2 ± 5.1	48.8 ± 5.8	<0.001	<0.001	0.02
FAZ area (mm^2^)	0.3 ± 0.2	0.3 ± 0.2	0.2 ± 0.1	0.31	NA	NA
FAZ perimeter (mm)	2.2 ± 0.7	2.1 ± 0.6	1.9 ± 0.5	0.35	NA	NA
Acirculatory index (AI)	1.1 ± 0.05	1.1 ± 0.04	1.1 ± 0.03	0.46	NA	NA
Foveal VD (FD-300) (%)	45.9 ± 7.4	51.7 ± 5.3	50.4 ± 7.3	0.01	0.01	0.007
Nonflow area (mm^2^)	0.6 ± 0.3	0.6 ± 0.2	0.5 ± 0.2	0.54	NA	NA

FAZ= foveal avascular zone; VD= vessel density.

p^*^= p-value for Kruskal-Wallis test; p^1^= p-value for two-group comparison of patients with Behcet disease with posterior uveitis and healthy controls; p^2^= p-value for two-group comparison of patients with Behcet disease with posterior uveitis and patients with Behcet disease without ocular involvement.

The VDs in patients with Behçet disease without ocular involvement were lower than those in the healthy controls, but this difference did not reach statistical significance (Table 2).

There was a moderate correlation between BCVA and parafoveal VD in the DCP (r=-0.62, p<0.001), perifoveal VD in the DCP (r=-0.43, p=0.006), and FD-300 (r=-0.42, p=0.008) (Table 3).

**Table 3:** Correlation between OCTA measurements and BCVA (logMAR) in patients with Behcet disease

	BCVA (logMAR)	
	r	p-value^*^
Superficial foveal VD	0.18	NS
Superficial parafoveal VD	-0.19	NS
Superficial perifoveal VD	-0.20	NS
Deep foveal VD	0.002	NS
Deep parafoveal VD	-0.62	<0.001
Deep perifoveal VD	-0.43	0.006
FAZ area	0.23	NS
FAZ perimeter	0.27	NS
Acirculatory index (Al)	0.23	NS
Foveal VD (FD-300)	-0.42	0.008
Nonflow area	0.18	NS

BCVA= best corrected visual acuity; NS= not significant; OCTA= optical coherence tomography angiography; VD= vessel density. p^*^= p-value for Pearson correlation analysis.

## DISCUSSION

Ocular involvement is a leading cause of morbidity in Behçet disease. Despite the advances in treatment, the estimated risk of loss of useful vision in patients with Behçet uveitis is 25% at 10 years^(15)^. The typical ocular manifestation is in the form of relapsing remitting nongranulomatous panuveitis with retinal vasculitis. Fluorescein angiography and OCT are currently the most commonly used methods in detecting and monitoring vascular lesion activity and associated secondary retinal complications in patients with Behçet disease^(2)^.

OCT-A is a novel, noninvasive imaging technique that has the ability to segment different layers of the retina and provide a detailed view of the retinal and choroidal vasculature for quantitative and qualitative analyses. While the inability of the current OCT-A technology to detect vascular leakage precludes its use as the sole diagnostic tool in uveitis follow-up, it is still a valuable tool that allows better visualization of microvascular alterations and can provide new objective biomarkers for ocular inflammation^(16-19)^.

In the present study, patients with Behçet uveitis showed reduced parafoveal and perifoveal VDs in the SCP and DCP compared to patients with Behçet disease without ocular involvement and healthy controls. Recently, Cheng et al.^(10)^ analyzed retinal vascular changes in patients with Behçet uveitis during remission. They found that superficial and deep capillary VDs were lower in patients with Behçet disease and the difference between patients and healthy controls was more prominent in the deep layer. In another study, Khairallah et al.^(11)^ evaluated OCT-A findings in eyes with active Behçet uveitis and found that capillary VD in the DCP was significantly lower in these patients. Areas of retinal capillary hypoperfusion, capillary abnormalities, and disorganization of the capillary network were also more frequent in the DCP compared to those in the superficial layer. The authors explained this predilection for more severe involvement in the deep retinal microvasculature by its location in a watershed-like region. The lack of such a differential involvement in our patient group can be explained by the differences in the study populations. A recent study involving patients with uveitis showed that VD in the deep plexus was significantly lower in subjects with uveitic macular edema^(19)^. We believe that the exclusion of patients with CME in our study might have caused selection bias toward patients with less severe involvement in the DCP.

FD-300, the foveal VD in the area 300 µm around the FAZ, is a relatively new OCT-A parameter that can be helpful in early detection of macular nonperfusion^(20)^. Unlike the foveal VDs measured by the density assessment tool of the software, FD-300 shows the VD at a distance of 300 µm around the FAZ and is not affected by FAZ diameter^(21)^. In the present study, the foveal VDs measured by the density assessment tool were similar between the groups. However, FD-300 was significantly lower in patients with Behçet uveitis. This finding suggests that changes in FD-300 can be a more valuable marker for detecting changes in macular perfusion.

Alterations in the FAZ can be an early predictor of macular ischemia. While the data from most OCTA studies in retinal vaso-occlusive diseases and diabetic retinopathy support this hypothesis, the findings in Behçet disease are contradictory^(7,22-24)^. Cheng et al.^(10)^ showed that the FAZ was enlarged in patients with Behçet uveitis with more than five attacks. However, Khairallah et al.^(11)^ were unable to detect a significant difference in the FAZ area between patients with Behçet disease and healthy controls. In the present study, the FAZ area and perimeter were measured in the full retina and larger in patients with Behçet disease, but this difference did not reach statistical significance. The high variability of the FAZ area within the population might have masked the changes in FAZ characteristics in our patient group.

In patients with Behçet disease, there was a significant correlation between BCVA and DCP parafoveal and perifoveal VDs (Table 3). A similar correlation between global and regional deep capillary VDs and BCVA was also reported in a previous study^(10)^. As a quantitative measure of the degree of macular ischemia, such a correlation between VD and visual acuity is understandable. Future longitudinal studies are required to ensure that these parameters can be used as predictors of visual outcome in Behçet uveitis.

Recent studies on patients with non-ocular Behçet disease have yielded encouraging results^(25,26)^. Rafaat et al.^(25)^ evaluated OCT-A findings in 10 patients with non-ocular Behçet disease and found that capillary density was significantly lower in patients with Behçet disease. Recently, Goker et al.^(26)^ showed that the FAZ area and perimeter were significantly higher in patients with Behçet disease without ocular involvement. Furthermore, SCP and DCP foveal VD and FD were significantly decreased in patients without Behçet disease. While the results of these studies suggest the possibility of a subclinical ocular involvement in patients with Behçet disease without any evidence of uveitis, our results contradict this hypothesis. VD measurements, FAZ characteristics, and nonflow area in the patients with Behçet disease without ocular involvement were similar to those in healthy controls in the present study. The longer disea se duration in the previous studies and difference in patient characteristics are the most likely explanations for this discrepancy.

The relatively small sample size and its cross-sectional design are the major limitations in our study. OCT-A image artifacts are common. Eyes with uveitis with poorer vision may have been more likely to be excluded due to inadequate scan quality resulting from the inability to fixate well during OCT-A acquisition. This may have led to study bias toward eyes with better visual acuity. The current algorithm in the AngioVue software does not allow evaluation of the middle capillary plexus (MCP). Future studies can focus on assessing the SCP, MCP, and DCP separately as they can show different levels of nonperfusion. The extramacular retina was not evaluated in the current study.

Therefore, we demonstrated a significant decrease in parafoveal VD, perifoveal VD, and FD in patients with Behçet uveitis. There was a significant correlation between visual acuity, parafoveal and perifoveal VD in the DCP, and FD. OCT-A may be a useful, noninvasive imaging modality to evaluate retinal microvascular involvement in patients with Behçet disease. The OCT-A findings in patients with Behçet disease without ocular involvement were similar to those in healthy controls. Further studies with a longitudinal follow-up including patients with/without ocular involvement are required to validate our findings and assess the role of reduced VD in evaluating Behçet disease-related ocular complications and predicting visual outcome.
